# Identification of an Oxidative Stress-Related LncRNA Signature for Predicting Prognosis and Chemotherapy in Patients With Hepatocellular Carcinoma

**DOI:** 10.3389/pore.2022.1610670

**Published:** 2022-10-05

**Authors:** Zixuan Zhong, Minxuan Xu, Jun Tan

**Affiliations:** ^1^ Chongqing Key Laboratory of Medicinal Resources in the Three Gorges Reservoir Region, School of Biological and Chemical Engineering, Chongqing University of Education, Chongqing, China; ^2^ Research Center of Brain Intellectual Promotion and Development for Children Aged 0-6 Years, Chongqing University of Education, Chongqing, China; ^3^ Department of Experimental Center, School of Biological and Chemical Engineering, Chongqing University of Education, Chongqing, China; ^4^ Key Laboratory of Biorheological Science and Technology, Ministry of Education, College of Bioengineering, Chongqing University, Chongqing, China

**Keywords:** hepatocellular carcinoma, survival analysis, immune cell infiltration, oxidative stress, drug sensitivity, lncRNA signature

## Abstract

**Background:** Oxidative stress plays a critical role in oncogenesis and tumor progression. However, the prognostic role of oxidative stress-related lncRNA in hepatocellular carcinomas (HCC) has not been fully explored.

**Methods:** We used the gene expression data and clinical data from The Cancer Genome Atlas (TCGA) database to identify oxidative stress-related differentially expressed lncRNAs (DElncRNAs) by pearson correlation analysis. A four-oxidative stress-related DElncRNA signature was constructed by LASSO regression and Cox regression analyses. The predictive signature was further validated by Kaplan–Meier (K–M) survival analysis, receiver operating characteristic (ROC) curves, nomogram and calibration plots, and principal component analysis (PCA). Single-sample gene set enrichment analysis (ssGSEA) was used to explore the relationship between the signature and immune status. Finally, the correlation between the signature and chemotherapeutic response of HCC patients was analyzed.

**Results:** In our study, the four-DElncRNA signature was not only proved to be a robust independent prognostic factor for overall survival (OS) prediction, but also played a crucial role in the regulation of progression and chemotherapeutic response of HCC. ssGSEA showed that the signature was correlated with the infiltration level of immune cells. HCC patients in high-risk group were more sensitive to the conventional chemotherapeutic drugs including Sorafenib, lapatinib, Nilotinib, Gefitinib, Erlotinib and Dasatinib, which pave the way for targeting DElncRNA-associated treatments for HCC patients.

**Conclusion:** Our study has originated a prognostic signature for HCC based on oxidative stress-related DElncRNAs, deepened the understanding of the biological role of four key DElncRNAs in HCC and laid a theoretical foundation for the choice of chemotherapy.

## Introduction

Hepatocellular carcinoma (HCC) is the most common malignant tumor of the liver and the fourth leading cause of cancer-related death worldwide. Chronic viral hepatitis, including hepatitis B virus (HBV) and hepatitis C virus (HCV), and nonalcoholic steatohepatitis (NASH) or cirrhosis are the most important risk factors for development of HCC [1], leading to enhanced ROS production and induction of oxidative stress in hepatocytes [2]. Currently, the effective treatments for HCC predominantly include targeted chemotherapy, hepatic resection, immunotherapy, and liver transplantation [3–5]. However, in spite of great advances in HCC treatment over the past decades, a large number of HCC patients still miss the best treatment opportunities due to late diagnosis, and the 5-year survival rate is unsatisfactory due to recurrence in a large proportion of patients after diagnosis at an advanced stage [6]. Thus, there is an urgent need to identify robust prognostic biomarkers for HCC patients who might benefit from curative therapy.

Oxidative stress occurs as a result of an imbalance between the generation of reactive oxygen species (ROS) and antioxidant molecules in cells, causing damage to DNA, lipids, and proteins [7]. ROS can promote cell proliferation, apoptosis, angiogenesis, tissue invasion and metastasis [8]. Oxidative stress has also been proposed to play a critical role in liver disease progression and liver carcinogenesis with overwhelming production of ROS. High levels of ROS can induce liver DNA injury and further progress to NASH, fibrosis, cirrhosis and HCC [9–11]. Excessive production of ROS during tumorigenesis creates an oxidative tumor microenvironment that in turn could be an important factor driving carcinogenesis and cancer progression [12, 13]. It has been reported that ROS promotes HCC progression by inducing nuclear factor erythroid 2-relatedfactor 2 (Nrf2) expression, 8-hydroxydeoxyguanosine (8-OHdG) formation and tumor progression in HCC cells [9, 14]. Although these studies have clarified that oxidative stress is closely correlated with HCC progression, the prognostic value of oxidative stress-related genes in HCC prognosis prediction is largely unclarified and the underlying mechanisms require further validation*.*

Long noncoding RNAs (lncRNAs) are noncoding molecular transcripts with over 200 nucleotides in length and involved in regulating the expression of various cancer-associated genes. Dysregulation of lncRNAs is widespread in cancer and has been reported to play essential roles in the progression of various human tumor [15]. To date, emerging evidences have shown that lncRNA can serve as biomarkers of tumor prognosis and diagnosis, such as gastric cancer, colorectal cancer and HCC [16–20]. Notably, a recent study has demonstrated that lncRNA affects the development of HCC by regulating oxidative stress [21]. According to the hypothesis, lncRNAs may regulate oxidative stress-related mRNA expression by acting as a competing endogenous RNA (ceRNA) to sponge miRNAs in HCC [22]. However, at present, the regulatory function of oxidative stress-related lncRNA remains unclear and the specific function of oxidative stress-related lncRNAs in HCC has not been fully elucidated.

In this study, we originally constructed an oxidative stress-related DElncRNA signature with internal verification, analyzed the biological role of four-DElncRNA signature in the overall survival of HCC patients, and evaluated the value of prognosis, diagnosis, chemotherapy response and tumor immune infiltration of HCC patients based on the lncRNA signature. Notably, the signature could be used as an independent prognostic biomarker for HCC without the need to consider other clinical variables and showed to be a promising therapeutic targets for HCC treatment.

## Materials and Methods

### Patients and Clinical Data Collection

The RNA transcriptome datasets and the associated clinical information of the HCC samples were downloaded from the Cancer Genome Atlas (TCGA) database (https://portal.gdc.cancer.gov/repository), which included 374 tumor samples and 50 normal tissue samples. Genes were grouped into protein coding genes and lncRNA genes based on the human genome annotation data by strawberry perl (version 5.30.0.1). The gene expression profiles were normalized using the scale method provided in the “limma” R package. To reduce statistical bias in this analysis, HCC patients with missing overall survival (OS) values or short OS values (<30 days) were excluded. With relevant clinical information, we finally retrieved 343 samples of HCC patients.

### Acquisition of Oxidative Stress-Related Genes and LncRNAs

807 oxidative stress protein domains were extracted from GeneCards (https://www.genecards.org) with a relevance score ≥7, and further preprocessed with the “limma” R package in view of a false discovery rate (FDR) < 0.05 and |log_2_ fold change (FC)| ≥1 to obtain 252 differentially expressed oxidative stress genes (DEOSGs) for further analysis. Likewise, differentially expressed lncRNAs (DElncRNA) were obtained with FDR <0.05 and |log_2_ FC|≥ 1 after screening the data matrix by “limma” R package. The identified DEOSGs in Gene Ontology (GO) analysis and Kyoto Encyclopedia of Genes and Genomes (KEGG) pathway enrichment analysis were performed with “clusterProfiler” R package. P and FDR values <0.05 were regarded as significantly different.

### Identification of Oxidative Stress-Related DElncRNAs

To identify oxidative stress-related DElncRNAs, correlation analysis was performed between DEOSGs and DElncRNAs by using an absolute correlation coefficient >0.4 and *p* value <0.001 as the screening criteria, a total of 1400 oxidative stress-related DElncRNAs with expression values were obtained.

### Construction of the DElncRNA-Based Prognostic Signature

The 343 complete samples of HCC patients were randomly classified into a training dataset (*n* = 207) and a testing dataset (*n* = 136) using the “caret” R package, with a ratio of 6:4. In the training dataset, the univariate Cox regression analysis was applied to identify the overall survival (OS)-related DElncRNAs (*p* < 0.001), which were subsequently used for the LASSO regression analysis. Then the regression coefficients of the prognostic signature were identified by a multivariate Cox regression model using “glment,” “survminer” and “survival” R packages. The risk score of HCC patients was calculated with the following computational equation: risk score = sum of coefficients × the DElncRNA expression. The risk score of HCC patients was computed in the training dataset, testing dataset, and the complete dataset. Subsequently, the patients were separated into high- and low-risk groups with the cutoff point set as the median values of the risk score of three datasets.

### Visualization of Oxidative Stress-Related LncRNA–mRNA Co-Expression

The oxidative stress-related DElncRNA–mRNA co-expression network was visualized using Cytoscape based on Pearson’s correlation. Sankey diagram of prognostic oxidative stress-related DElncRNAs was visualized with “ggalluvial” R package.

### Survival, ROC and PCA Analysis

With “survival” and “survminer” R packages, the Kaplan–Meier (K-M) survival curve analysis was performed on the OS rates in the training dataset, testing dataset and the complete dataset. Then, the “timeROC” package was also used for receiver operating characteristic (ROC) curve analysis. The area under the curve (AUC) value was calculated for the evaluation of the predicted accuracy of oxidative stress-related DElncRNA prognostic signature. Principal component analysis (PCA) analysis was used to further validate the feasibility of the DElncRNA signature model in HCC patients with “limma” and “scatterplot3d” packages.

### Nomogram and Calibration

With “Survival”, “regplot” and “rms” R packages, the oxidative stress-related DElncRNA signature-based risk score and independent clinical factors were used to set up a nomogram for the 1-, 3-, and 5-year OS and correction curves to validate the calibration and accuracy of the nomogram.

### Gene Set Enrichment Analyses

With the curated gene set (c2.kegg.v7.5.symbols.gmt), gene set enrichment analyses (GSEA) software (https://www.gsea-msigdb.org/gsea/login.jsp) was applied to identify the significantly enriched KEGG pathways between the low-risk and high-risk groups based on the criterion:|NES|>1, *p* < 0.05 and FDR <0.25. The graph was then constructed by the “ggplots” and “gridExtra” R packages. The infiltration scores of 16 immune cells and the activities of 13 immune-related functions were calculated by single-sample gene set enrichment analysis (ssGSEA) using the “GSVA” R package.

### Prediction of Chemotherapeutic Response

The sensitivity of anticancer drugs was evaluated by gene expression levels of HCC patients based on the Cancer Genome Project (CGP) using “pRRophetic” R package [23]. The correlation of DElncRNA expression in the signature model and activity of FDA-approved drugs in the NCI60 panel of human tumor cell lines were evaluated using CellMiner database (https://discover.nci.nih.gov/cellminer/home.do)[24].

### Tumor Mutation Burden Analysis

The single nucleotide variation (SNV) data of HCC patients were downloaded from TCGA database. The somatic variant data was stored as Mutation Annotation Format (MAF), and the maftools were used to analyze the mutation data of HCC samples and calculate the Tumor mutation burden (TMB) scores of HCC patients [25]. K-M analysis was used to analyze the survival rate between patients with a high TMB and risk score and patients with a low TMB and risk score.

### Statistical Analysis

All computational and statistical analyses were performed using R (v4.0.5) software (https://www.r-project.org/). The Wilcoxon test was used to analyze the expression levels of DEOSGs in tumor and normal tissues. For all analyses, *p* values < 0.05 were regarded as statistically significant.

## Results

### Identification of Oxidative Stress-Related DElncRNA

**FIGURE 1:**
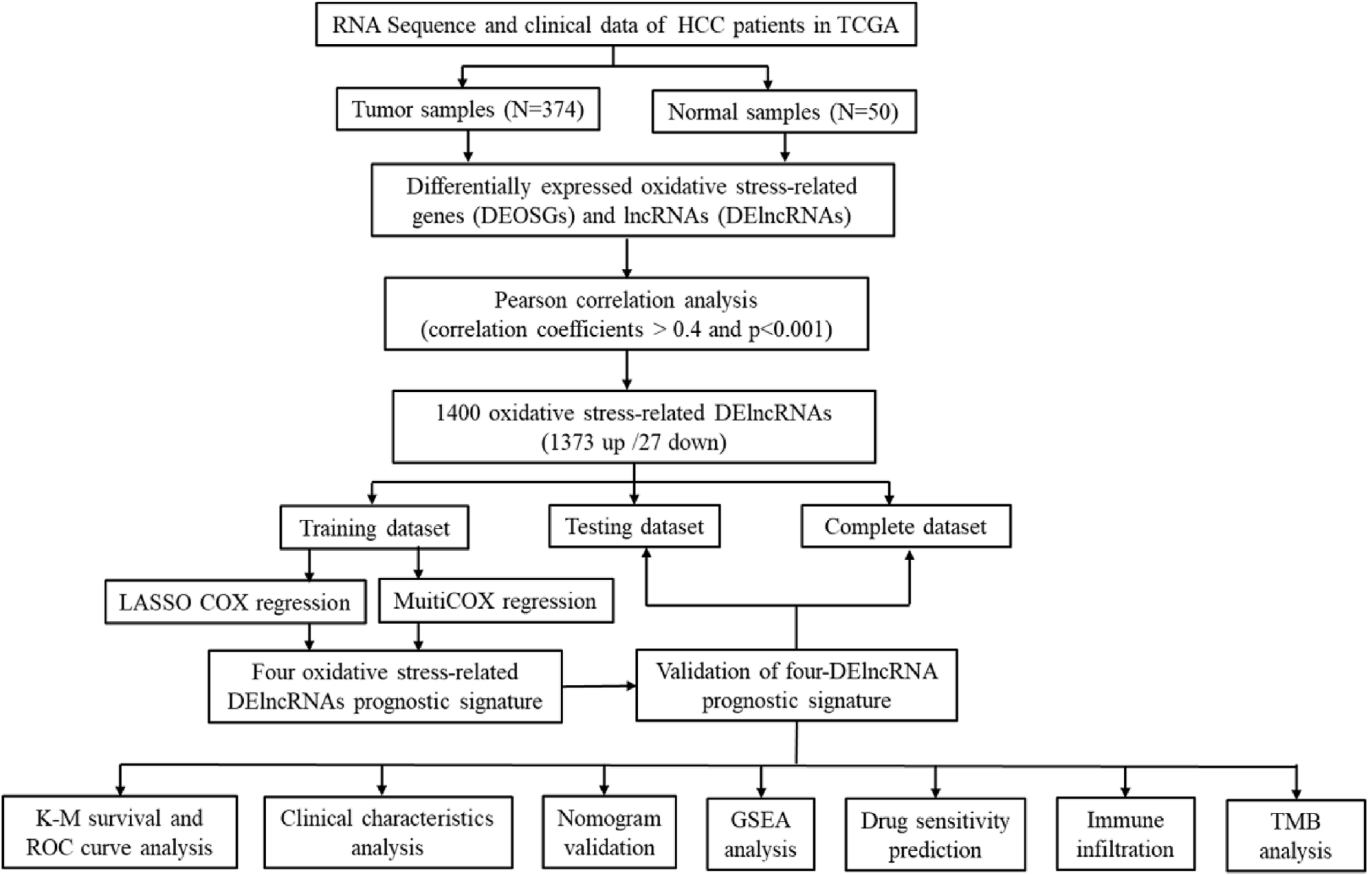
Flow chart of this study.

The workflow of the study was displayed in Figure 1. The oxidative stress-related genes were obtained from GeneCards with a relevance score ≥7, and the GO and KEGG pathway enrichment analysis of DEOSGs were shown in Supplementary Figure S1. According to the expression of 252 DEOSGs and 3,382 DElncRNAs (FDR <0.05 and |log_2_FC|≥1) between normal and tumor samples, 1,400 oxidative stress-related DElncRNAs were identified with an absolute correlation coefficient |R^2^ | > 0.4 and *p* < 0.001. Of them, 1,373 DElncRNAs were upregulated, and only 27 were downregulated (Supplementary Figure S2).

### Construction of Oxidative Stress-Related DElncRNA Prognostic Signature

**FIGURE 2:**
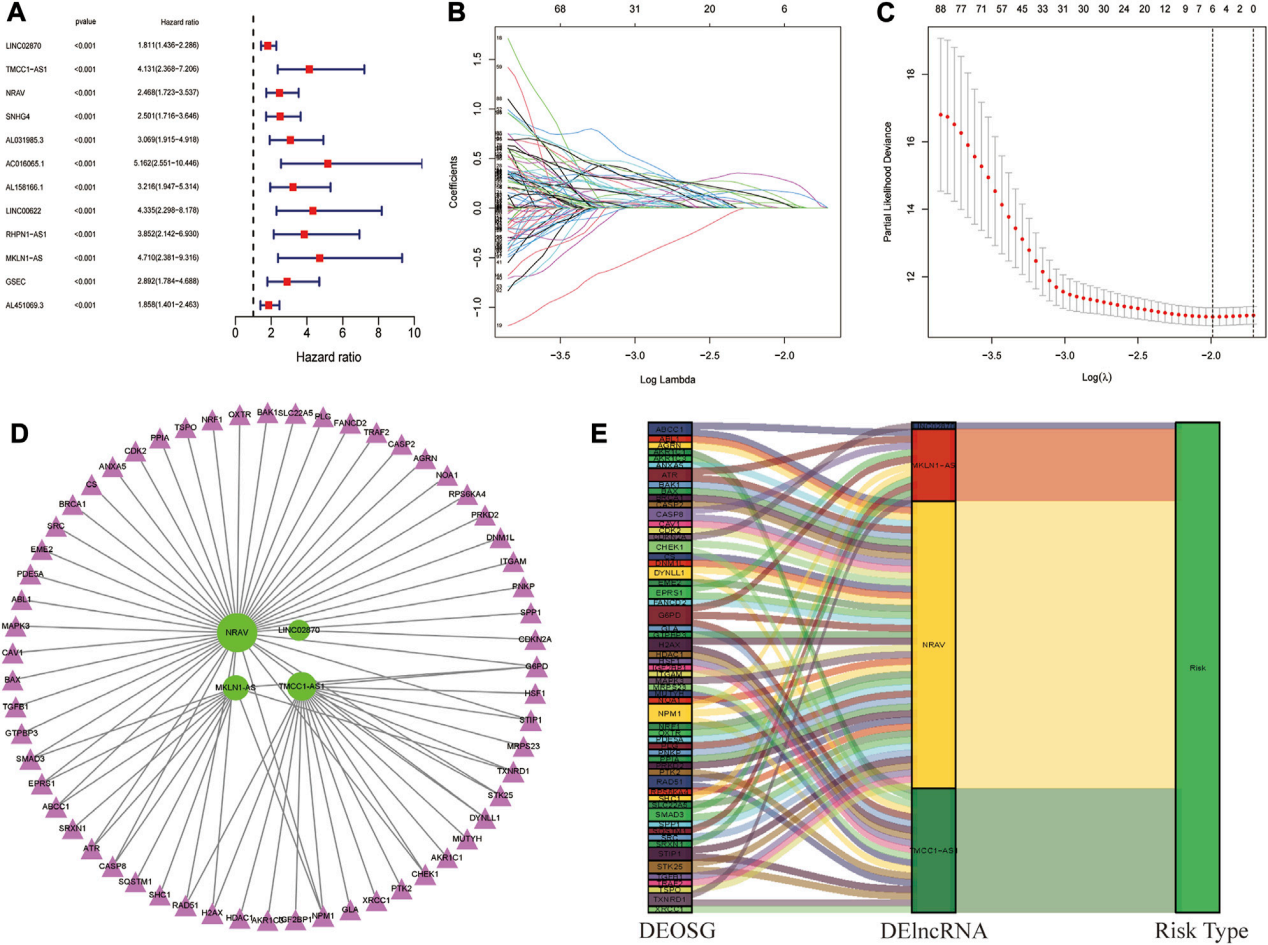
Identification of oxidative stress-related DElncRNA signature in HCC. **(A)** Top 12 out of 64 prognostic DElncRNAs with Hazard ratio and *p* values extracted by univariate Cox regression analysis (all *p* values < 0.001). **(B)** The 10-fold cross-validation for variable selection in the LASSO model. **(C)** The LASSO coefficient profile of six oxidative stress-related DElncRNAs. **(D)** Coexpression network of four prognostic oxidative stress-related DElncRNAs and DEOSGs. Green ellipses indicate prognostic DElncRNA signature, and pink triangles indicate DEOSGs. **(E)**The Sankey diagram of four oxidative stress-related DElncRNAs, DEOSGs, and risk type.

A total of 343 complete HCC samples were randomly grouped into a training dataset (*n* = 207) and a testing dataset (*n* = 136), with a ratio of 6:4. According to a univariate Cox regression analysis, 64 oxidative stress-related DElncRNAs significantly correlated with overall survival (OS) (*p* value <0.001) (Figure 2A, Supplementary Table S1). Next, the LASSO regression model analysis was employed for verifying further variables by10-fold cross-validation and six key DElncRNAs were produced in this progress (Figures 2B,C). Finally, four DElncRNAs (LINC02870, TMCC1-AS1, NRAV and MKLN1-AS) were screened to construct a prognostic signature for HCC patients though multivariate Cox regression analysis. The co-expression network of the four DElncRNAs and DEOSGs (|R^2^ | > 0.4 and *p* < 0.001) were visualized with Cytoscape (Figure 2D), which showed total 75 pairs DElncRNAs-DEOSGs. Moreover, all this four DElncRNAs (LINC02870, TMCC1-AS1, NRAV and MKLN1-AS) were risk factors for OS (Figure 2E). The risk score for each patient in the training dataset, testing dataset, and the complete dataset was calculated based on the risk formula: risk score = LINC02870 × 0.373 + TMCC1-AS1 × 0.777 + NRAV × 0.384 + MKLN1-AS × 0.843 (Supplementary Table S3).

### Prognostic Value of Oxidative Stress-Related DElncRNA Signature

**FIGURE 3:**
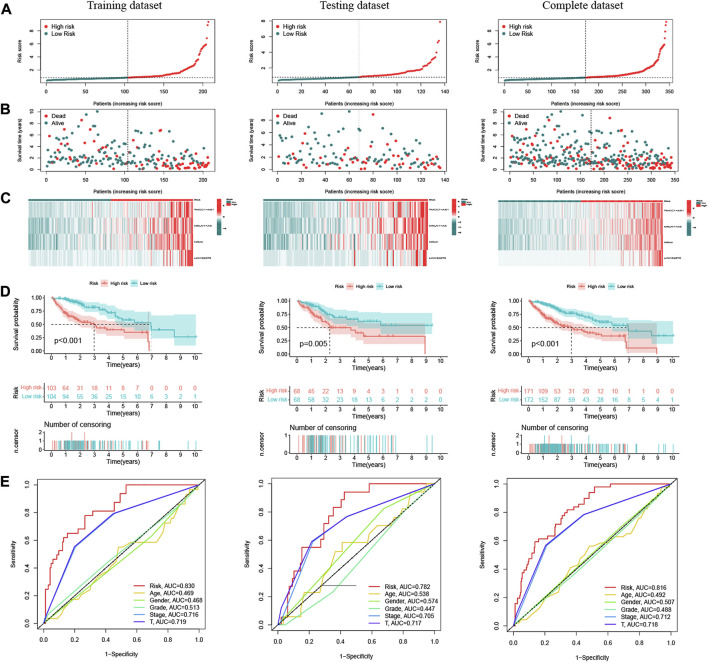
Prognostic analysis of oxidative stress-related DElncRNA-based signature model in training, testing and complete datasets. **(A)** Distribution of patients’ risk scores. **(B)** Patients’ overall survival (OS) status distribution. **(C)** Prognostic signature heatmaps. **(D)** K-M survival curves of HCC patients in high-risk and low-risk groups. **(E)** Time-dependent ROC curve analyses for predicting OS by risk score, age, sex, stage, T stage.

Patients in the training dataset, testing dataset and complete dataset were further grouped into a high-risk group and low-risk group based on the median risk score. The distributions of patients’ risk score, OS status and DElncRNA signature expression in the three datasets were shown in Figures 3A–C, respectively, which all illustrated more death cases were in the high-risk group with the increase of risk score. K–M survival curve indicated that the low-risk patients had a better prognosis than high-risk patients in the training dataset(*p* < 0.001), testing dataset (*p* = 0.005) and complete dataset (*p* < 0.001), respectively (Figure 3D). Time-dependent ROC analysis showed that the AUC of the risk score predicted OS was 0.830 in the training dataset, 0.782 in the testing dataset, and 0.816 in the complete dataset, respectively (Figure 3E), and AUC at 1, 3, and 5 years in the three datasets also indicated a good OS rate prediction (Supplementary Figure S3). All these results implied that the four oxidative stress-related DElncRNA signatures would be a valuable HCC prognostic model.

### PCA Validation of the Stratification Capacity of DElncRNA Signature

**FIGURE 4:**
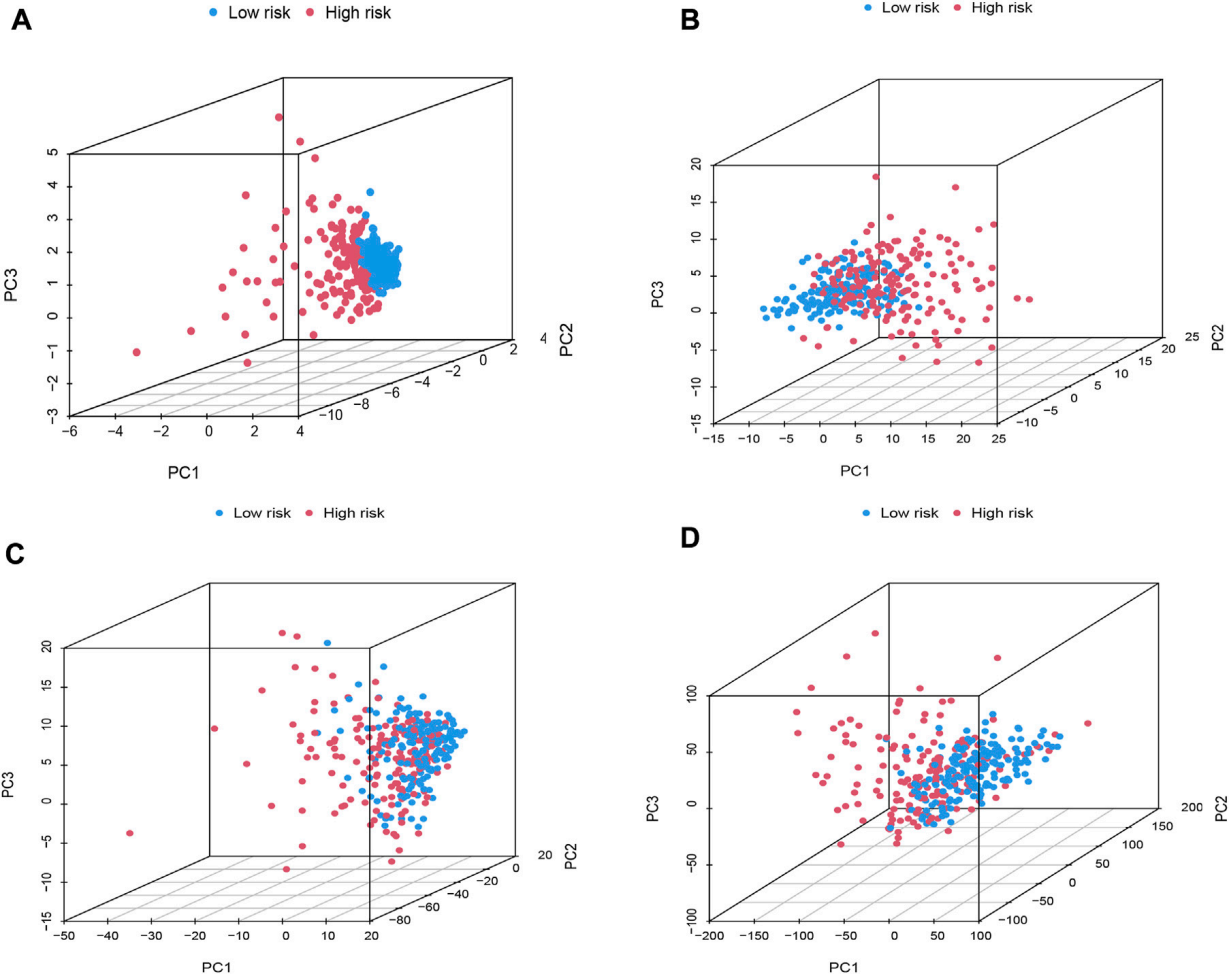
PCA plot of high-risk and low-risk groups based on **(A)** prognostic signature model including four oxidative stress-related DElncRNAs, **(B)** differentially expressed oxidative-related genes, **(C)** oxidative stress-related DElncRNAs, and **(D)** the entire human gene expression profiles.

The PCA was further performed to distinguish between high-risk group and low-risk group based on oxidative stress-related DElncRNA signature in training, testing and complete datasets (Supplementary Figure S4), which showed HCC patients were divided into high-risk and low-risk groups with a relatively clear resolution in all three datasets. According to the 3D PCA results in Figures 4A–D, there was a good heterogeneity in high-risk and low-risk groups based on the signature (Figure 4A), and the scattered distributions of high-risk and low-risk groups in Figures 4B–D also confirmed the potency of our DElncRNA-based prognostic signature in the discriminating high-risk and low-risk groups.

### Correlation Between the DElncRNA Signature Model and Clinical Factors

**FIGURE 5:**
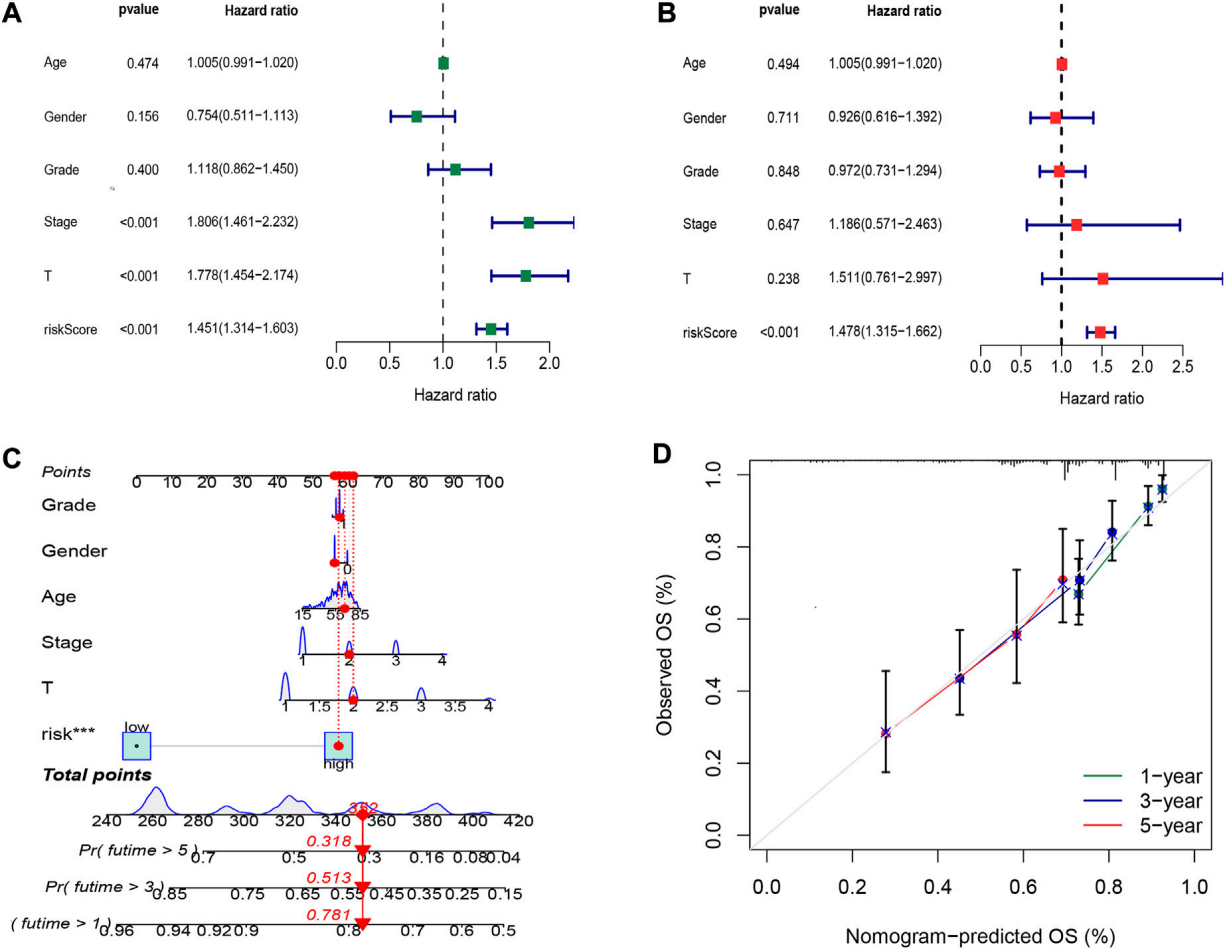
Cox regression analysis and nomogram prediction of HCC patients. **(A)** Forest plot of the overall survival (OS) prognostic values through univariate Cox regression analysis. **(B)** Forest plot of the OS prognostic values through multivariate Cox regression analysis. **(C)** Nomogram of the prognostic model based on four DElncRNA signatures and clinical factors. **(D)** Calibration curve analysis of the 1-, 3-, and 5-year survival prediction accuracy of the nomogram.

**FIGURE 6:**
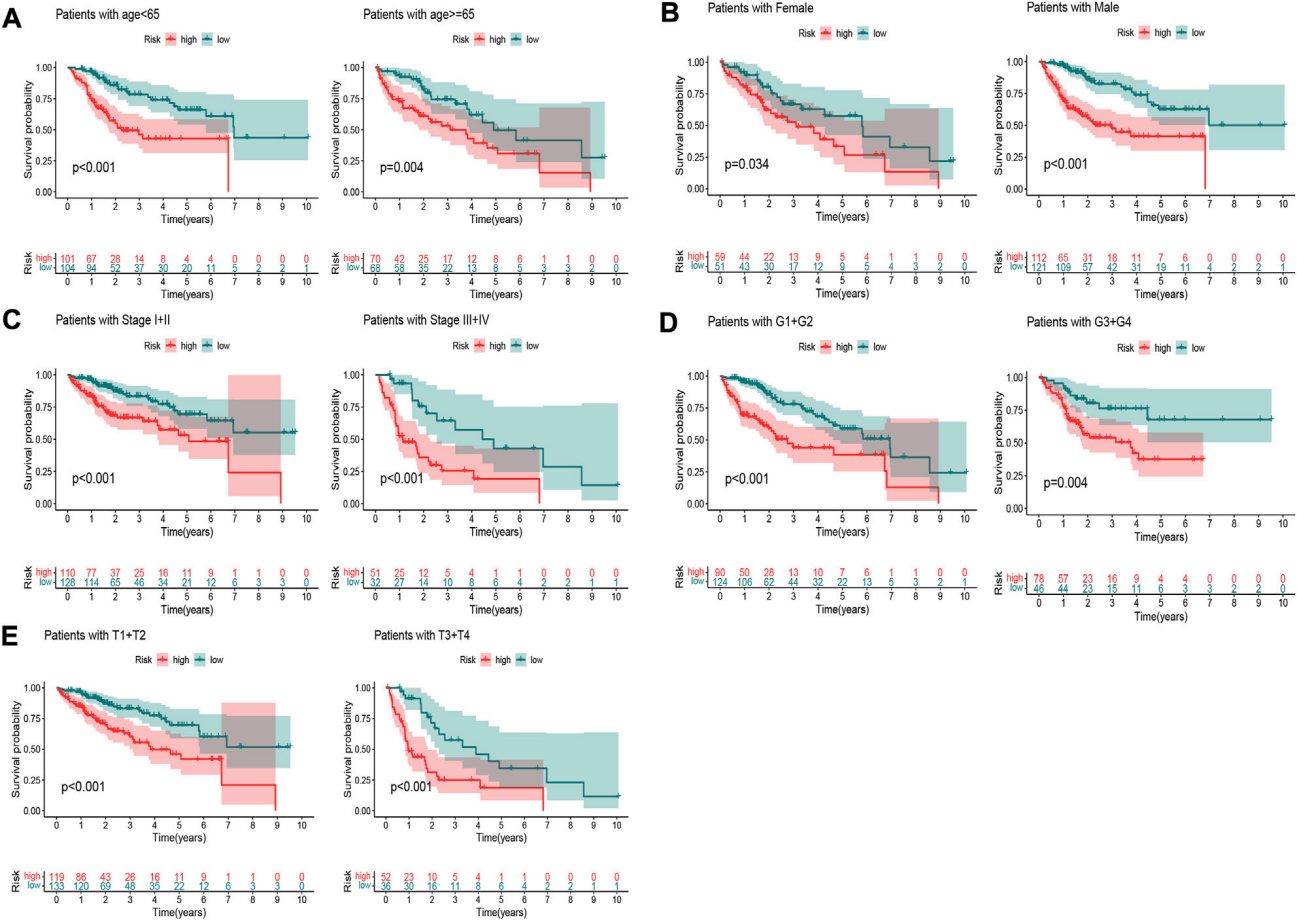
K–M survival curves of patients in subset groups. **(A)** Age <65 years and ≥65 years; **(B)** Males and females; **(C)** Stage I+II and stage III+IV; **(D)** G1+G2 and G3+G4; **(E)** T1+T2 and T3+T4.

To explore the oxidative stress-related DElncRNA signature affecting the prognosis of HCC patients, univariate and multivariate Cox regression analyses of OS-related clinical factors in the complete dataset were performed. The univariate Cox regression analysis showed that clinical stage, T stage and risk score were significantly related to the prognosis of HCC patients (Figure 5A, *p* < 0.001), and multivariate Cox regression analysis also showed the risk score was significantly associated with the prognosis of HCC patients (Figure 5B, *p* < 0.001). To make the DElncRNA-based signatures more applicable in the clinic, a nomogram with the risk score and clinical factors was built to predict the 1-, 3-, and 5-year OS rate of HCC patients (Figure 5C), and it implied patients with a higher total score represented a low OS rate. Then, a calibration curve was constructed to evaluate the consistency between the predicted OS rate by the nomogram and the actually observed OS rates, and it showed relatively good fit for the 1-, 3-, and 5-year OS prediction (Figure 5D). In addition, the results of K-M survival analysis were shown as subset groups separated by age, gender, clinical stage, grade and T stage (Figures 6A–E, respectively), which indicated OS rates in the high-risk group associated with age, gender, clinical stage, grade and T stage were significantly lower than that in low-risk group.

### GSEA of DElncRNA Signature-Based Pathways

**FIGURE 7:**
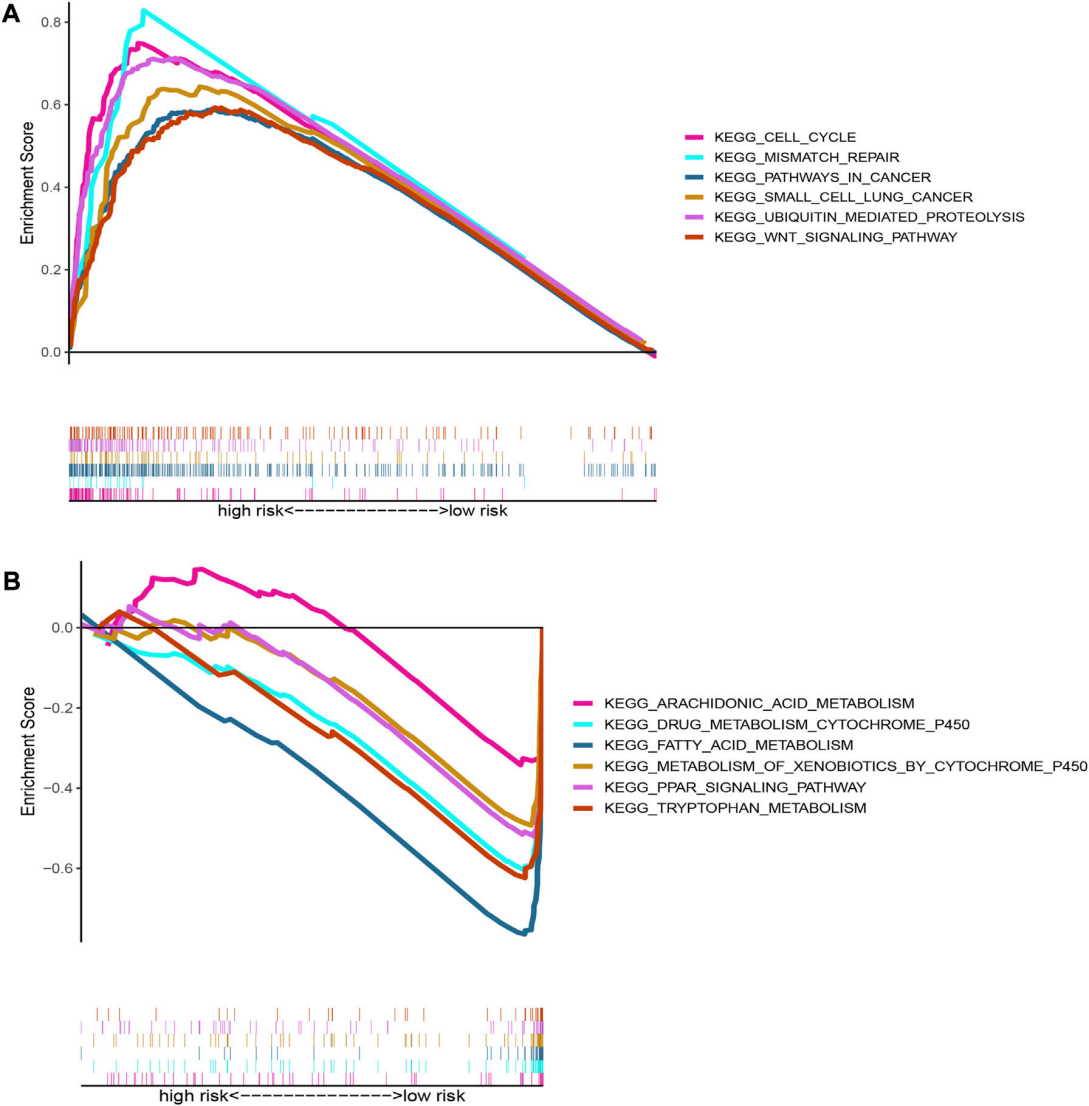
Pathway enrichment plots of GSEA based on oxidative stress DElncRNA signature. **(A)** Top six Kegg pathways enriched in high-risk group; **(B)** Top six Kegg pathways enriched in low-risk group.

The GSEA was performed to investigate differences in functions or pathways between high-risk group and low-risk group according to oxidative stress-related DElncRNA signature, and the six top significantly enriched KEGG pathways in high-risk and low-risk groups (|NES|>1, *p* < 0.05 and FDR <0.25) were showed in Figures 7A,B, respectively. The pathways of cell cycle, mismatch repair, pathways in cancer, small cell lung cancer, ubiquitin mediated proteolysis and WNT signaling pathway were significantly enriched in high-risk group, whereas pathways of arachidonic acid metabolism, drug metabolism, PPAR signaling pathway, fatty acid metabolism, xenobiotics metabolism and tryptophan metabolism significantly enriched in low-risk group. These GSEA results indicated that the DElncRNA signature may be associated with antitumor activity and sensitivity of chemotherapeutic agents.

### Analysis of DElncRNA Signature-Based Drug Sensitivity

**FIGURE 8:**
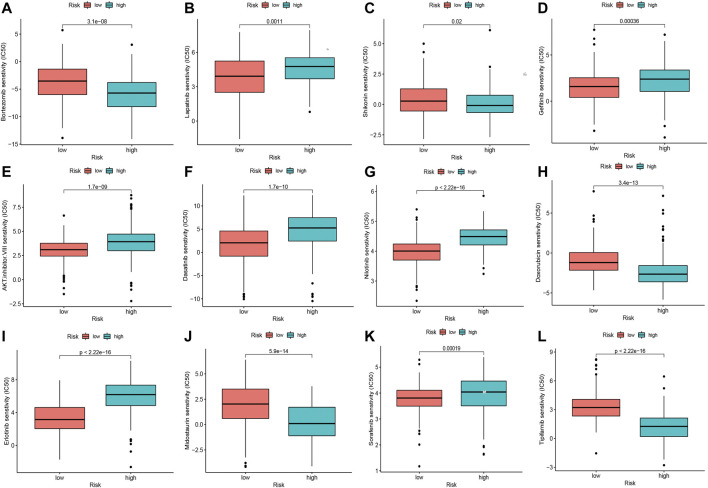
Significant sensitivity (calculated IC50) differences of 12 anticancer drugs between high-risk and low-risk groups. **(A)** Bortezomib, **(B)** Lapatinib, **(C)** Shikonin, **(D)** Gefitinib, **(E)** AKT. Inhibitor.VIII, **(F)** Dasatinib, **(G)** Nilotinib, **(H)** Doxorubicin, **(I)** Erlotinib, **(J)** Midostaurin, **(K)** Sorafenib and **(L)** Tipifarnib. (IC50, half-maximal inhibitory concentration).

To explore the potential drugs with antitumor activity for HCC treatment, the association between the oxidative stress-related DElncRNA predictive signature and the efficacy of anticancer drugs was analyzed. The results indicated that the sensitivity of sorafenib, nilotinib, lapatinib, gefitinib, erlotinib, dasatinib, and AKT inhibitor VIII were higher in high-risk group, whereas the sensitivity of bortezomib, doxorubicin, midostaurin, shikonin and tipifarnib were higher in low-risk group (Figure 8), which is valuable for exploring individualized chemotherapy for HCC patients in high-risk and low-risk groups. Moreover, among this four DElncRNAs, NRAV and TMCC1-AS1 are the two most sensitive lncRNAs for drugs with antitumor activity (Supplementary Figure S5, Supplementary Table S2).

### Analysis of DElncRNA Signature-Based Tumor Mutation Burden

**FIGURE 9:**
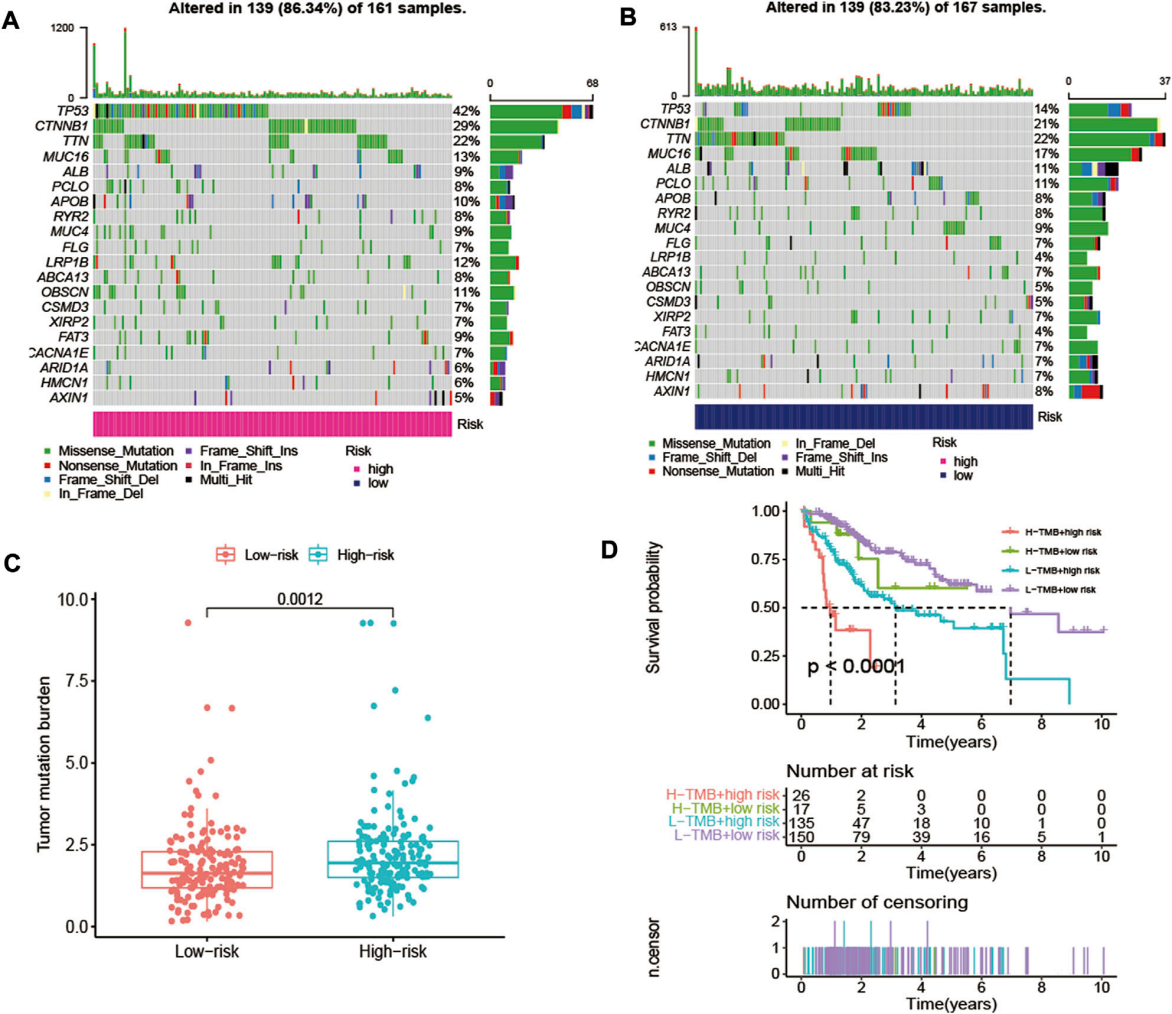
The differences of TMB in high-risk and low-risk groups of HCC patients. **(A)** The SNV alteration in high-risk group. **(B)** The SNV alteration in low-risk group. **(C)** Comparation of TMB in high-risk and low-risk groups. **(D)** The K-M survival curves of HCC patients in high-risk and low-risk groups with high TMB or low TMB.

The TMB has been demonstrated to be a useful biomarker for effective prediction of immune checkpoint blockade activity [26]. The SNV in high-risk and low-risk groups of HCC patients were evaluated, and the SNV data of 161 high-risk HCC samples and 167 low-risk HCC samples were obtained (Figures 9A,B). As a result, it could be noted that higher TMB in the high-risk group than that in low-risk group of HCC patients (Figure 9C). The OS rate of high-risk and low-TMB group was better than that of the high-risk and high-TMB group (*p* < 0.0001) (Figure 9D).

### Estimation of Immune Cell Infiltration and Immune-Related Function

**FIGURE 10:**
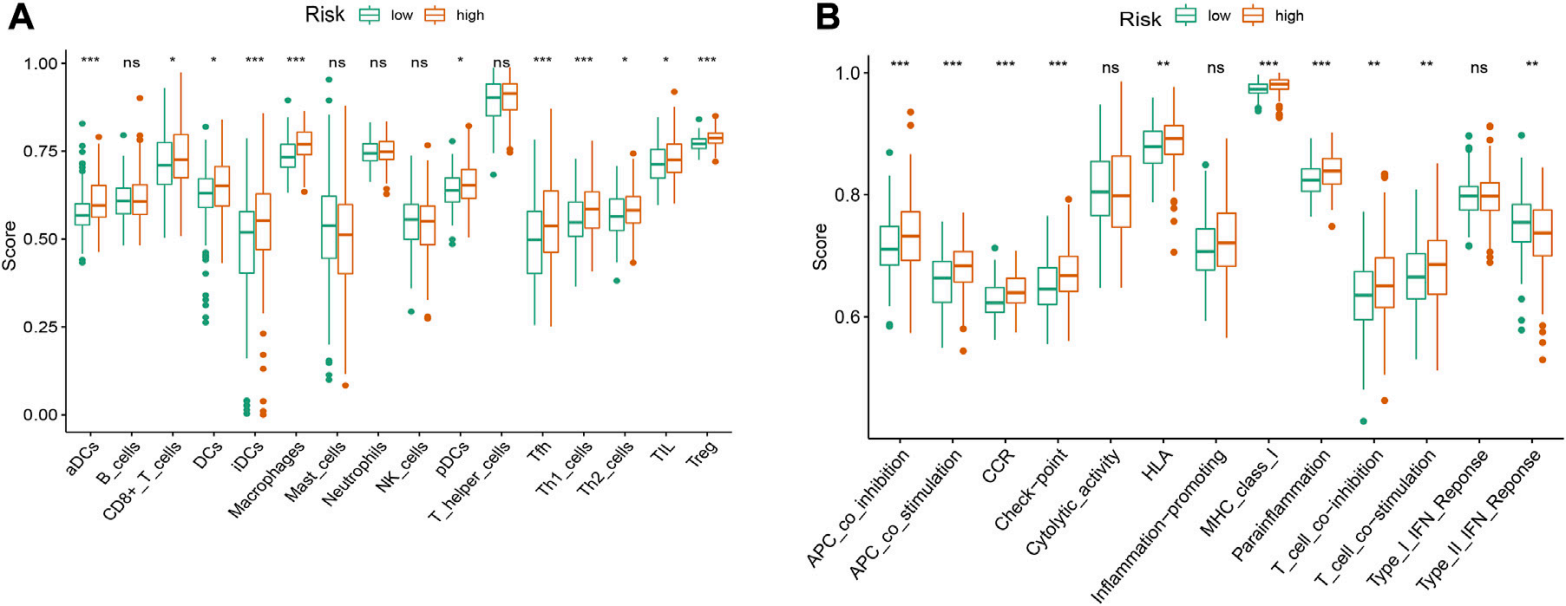
The ssGSEA scores of immune infiltrating cells and immune-related functions in high- and low-risk groups of HCC patients. **(A)** Comparison of infiltration levels of immune cells between high-risk and low-risk groups. **(B)** Comparison of immune-related functions between high-risk and low-risk groups. aDCs, activated dendritic cells; iDCs, immature dendritic cells; NK, natural killer; pDCs, plasmacytoid dendritic cells; Tfh, T follicularhelper; Th1, T helper type 1; Th2, T helper type 2; TIL, tumor-infiltrating lymphocyte; Treg, T regulatory cell; APC, antigen-presenting cell; CCR, chemokine receptor; HLA, human leukocyte antigen; MHC, major histocompatibility complex; IFN, interferon. *p < 0.05; **p < 0.01; ***p < 0.001; ns, non-significant.

To evaluate immune cell infiltration, ssGSEA was used to quantify the enrichment scores of tumor-infiltrating immune cell subgroups and immune-related function. The results showed that ssGSEA scores of activated dendritic cells (aDCs), immature dendritic cells (iDCs), macrophages, T helper cells, T follicular helper (Tfh) cells, T helper type 1 (Th1) cells and T regulatory cells (Tregs) were significantly different between the high-risk and low- risk groups (Figure 10A, *p* < 0.001). The immune function scores of antigen-presenting cell (APC) coinhibition, APC costimulation, chemokine receptor (CCR), checkpoint, major histocompatibility complex (MHC) class I and parainflammation were significantly different between the high-risk and low- risk groups (Figure 10B, *p* < 0.001).

## Discussion

Oxidative stress has been known to play a critical role in HCC progression [2, 9, 14, 27]. In our study, GO and KEGG pathway enrichment analysis of DEOSGs in HCC patients also indicated that DEOSGs were mainly involved in MAPK/TNF signaling pathway, chemical carcinogenesis, hepatitis B and nonalcoholic fatty liver disease (Supplementary Figure S1). LncRNAs can serve as diagnostic or prognostic biomarkers for many cancers [28–31], including HCC [16, 19, 20] and recently were reported to be involved in the response to oxidative stress [22, 32], however, there is no study on oxidative stress–lncRNA combinations in prognostic prediction for cancer patients, and the role of oxidative stress related-lncRNAs in the prognosis and diagnosis of HCC remains to be clarified. Thus, further exploring the regulatory function of oxidative stress-related lncRNAs in the prognosis of HCC will contribute to a better understanding of the molecular mechanism of HCC progression and help to identify reliable and effective biomarkers for HCC prognosis.

Here, in the present study, we for the first time constructed an oxidative stress-related four-DElncRNA signature, including LINC02870, TMCC1-AS1, NRAV and MKLN1-AS, all of them were significantly upregulated in the high-risk groups of HCC patients. Notably, LINC02870, TMCC1-AS1 and NRAV were also the top three significant hazard factors according to our univariate Cox regression analysis results. In addition, these four DElncRNAs are indicated by several independent bioinformatics analyses to have prognostic value in HCC [33–36]. However, studies on the prognostic value of HCC in relation to oxidative stress are still lacking for these four lncRNAs. In the coexpression network and sankey diagram of the DElncRNA signature and oxidative stress-related genes (Figure 2), TMCC1-AS, NRAV and MKLN1-AS were all positively correlated with Nucleophosmin 1 (NPM1). NPM1 is overexpressed in several types of tumors and has been evidenced to promote the occurrence and progression of tumors [37, 38], currently it could be used as a prognostic biomarker involved in immune infiltration of lung adenocarcinoma (LUAD) [39]. TMCC1-AS and NRAV were both positively correlated with RAD51 and STIP1. RAD51 is highly expressed in a large proportion of cancers, enhanced RAD51 expression is associated with a poor prognosis and reduced response to treatment [40]. STIP1 has also been suggested to be used as a prognostic biomarker for cancer treatment based on its high expression significantly associated with shorter OS [41]. LINC02870 was only positively correlated with CDKN2A, which is an essential regulator of immune cells and currently is used as a prognostic biomarker for determining prognosis and immune infiltration in HCC [42]. The occurrence and progression of the tumor were usually companied by oxidative stress, so these oxidative stress-related genes (including NPM, RAD51, STIP1and CDKN2A) may be involved in multiple mechanisms in HCC and play various biological functions associated with tumorigenesis, and their specific regulation mechanisms still need further study. As the four DElncRNAs may regulate the expression of these important oxidative stress-related genes, providing promising directions for clarifying the underlying molecular mechanisms of the DElncRNA signature for HCC progression*.*

Notably, the four-DElncRNA prognostic signature could efficiently stratify OS outcome of HCC patients between high-risk and low-risk groups in training and testing datasets. Moreover, the DElncRNA signature also showed ideal distinctions of OS of patients within different clinical subgroups. Further, ROC analysis results suggested that the signature has a high accuracy in OS rate prediction of HCC patients in training and testing datasets, and the AUC value is higher than other lncRNA-based prognostic signatures of HCC in previous reports [36, 43]. These results demonstrated that our DElncRNA signature has a good prognostic value. In addition, the GSEA results revealed that patients in the high-risk group were associated with enhanced tumorigenesis-related pathways, including pathways in cancer cell cycle, and WNT signaling pathway. HCC patients in the low-risk group were associated with intensified metabolic pathways related to anti-inflammation and anti-tumorigenesis, including arachidonic acid metabolism, drug metabolism, PPAR signaling pathway, fatty acid metabolism, xenobiotics metabolism and tryptophan metabolism, providing new evidence for supporting that the four DElncRNAs could serve as an efficient biomarker for HCC.

The biological functions of four DElncRNAs in the pathogenesis of HCC were just preliminarily studied recently. LINC02870 could promote tumor progression in HCC by inducing SNAIL translation [44]. TMCC1-AS1 employs an oncogenic role in promoting the proliferation, migration, invasion and epithelial-mesenchymal transition process of liver cancer cells [45]. Similarly, MKLN1-AS also could intensify proliferation, migration and invasion of HCC cells via Yes-associated transcriptional regulator 1 (YAP1) [46]. NRAV has been reported to be as a key regulator of antiviral immunity and involved in immunity response of HCC [36, 47]. In this study, the sensitivity of the four-DElncRNA signature to anticancer drugs in HCC patients were further investigated. The results suggested that patients in high-risk group were associated with higher sensitivity to anticancer drugs such as Sorafenib, Lapatinib, Nilotinib, Gefitinib, Erlotinib and Dasatinib. Among of them, Sorafenib is an anticancer drug approved by US-FDA for the preferred treatment of advanced HCC with the function of suppressing tumor cell proliferation [48, 49], and our result is different from the report in W Hong and L Liang et al. [50] which failed to find difference in the sensitivity of Sorafenib between high-risk and low-risk groups of HCC. Other drugs have also been applied in clinical trials for the treatment of different cancers, like lapatinib used in breast cancer [51], Nilotinib used in breast cancer and myeloid leukemia [52, 53], Gefitinib and Erlotinib used in lung cancer [54, 55], and Dasatinib used in myeloid leukemia [56], which provide a promising strategy with clinical implication for guiding individual and combined chemotherapy for HCC treatment.

Besides, the influence of differential expression of four DElncRNAs on the effect of different chemotherapeutic agents in the NCI60 panel of human tumor cell lines were explored in our study, and the results showed that only the expressions of TMCC1-AS and NRAV were significantly correlated with the activity of candidates of FDA-approved drugs (Supplementary Table S2, *p* < 0.05). TMCC1-AS expression was negatively correlated with the therapeutic effect of Bortezomib, Carboplatin, Tepotinib and Vorinostat. NRAV expression was negatively correlated with the therapeutic effect of Vandetanib, Erlotinib and Everolimus. These drugs were all reported to be effective chemotherapeutic agents for HCC treatment in previous clinical practices [57–62]. Thus, the results also implied that TMCC1-AS and NRAV maybe play a crucial role in the chemotherapy of HCC patients by promoting tumor progression and exhibiting a resistant response to these anticancer agents*.*

It has been known that immune cells play an essential role in interconnecting oxidative and inflammatory stress and modulating the rate of cell aging [63]. Moreover, immune cell infiltration can regulate tumor progression and affect prognosis of patients with cancer [64, 65]. Notably, our signature model demonstrated that the risk score was positively correlated with the infiltration levels of certain immune cells, including aDCs/iDCs/DCs, CD8^+^ T cells, Mccrophages, Tfh, Th1/Th2 cells, TIL and Treg, indicating that oxidative stress-related DElncRNA signature might serve as a key role in immune cell infiltration in HCC. Previous studies have shown that high infiltration of CD8^+^ T cells, Tregs and tumor-associated macrophages were associated with a poor prognosis in patients with different cancers [66–68]. In addition, the high TMB has been suggested to be correlated with the curative effect of immune checkpoint inhibitor treatments, and our result showed high-risk group had more TMB in tumor cells compared to low-risk group and HCC patients with high TMB had a poor prognosis, which was consistent with the conclusion of previous studies [69, 70].

However, large-scale cohort studies are still needed to further evaluate the stability of this signature model, and the underlying mechanism of this DElncRNA signature regulating its associated DEOSGs should be studied in depth as well.

## Conclusion

In conclusion, this study established a novel oxidative stress-related four-DElncRNA signature with a high prognostic value and can serve as an independent prognostic factor for HCC. To the best of our knowledge, this is the first report to develop an oxidative stress-related lncRNA signature model for HCC. Our study elucidated the important role of the signature in the prognosis and chemotherapeutic agent response of HCC and found several crucial oxidative stress-related genes that maybe correlated with HCC prognosis. The results also suggested that the signature might be a crucial mediator of immune function associated with oxidative stress in the tumor environment, representing promising therapeutic targets for improving the immunotherapeutic and chemotherapeutic efficacy of HCC and providing a theoretical basis for the development of personalized therapy of HCC.

## Data Availability

The data of this study are from TCGA databases.
