# Supplementary material for: Identification of an Oxidative Stress-Related LncRNA Signature for Predicting Prognosis and Chemotherapy in Patients With Hepatocellular Carcinoma
Source: Pathol Oncol Res. 2022 Oct 5;28:1610670. doi: 10.3389/pore.2022.1610670 (PMC9579291; doi:10.3389/pore.2022.1610670)
Supplement: Supplementary file 3 [file DataSheet1.PDF]

Supplementary Information

Supplementary Figures

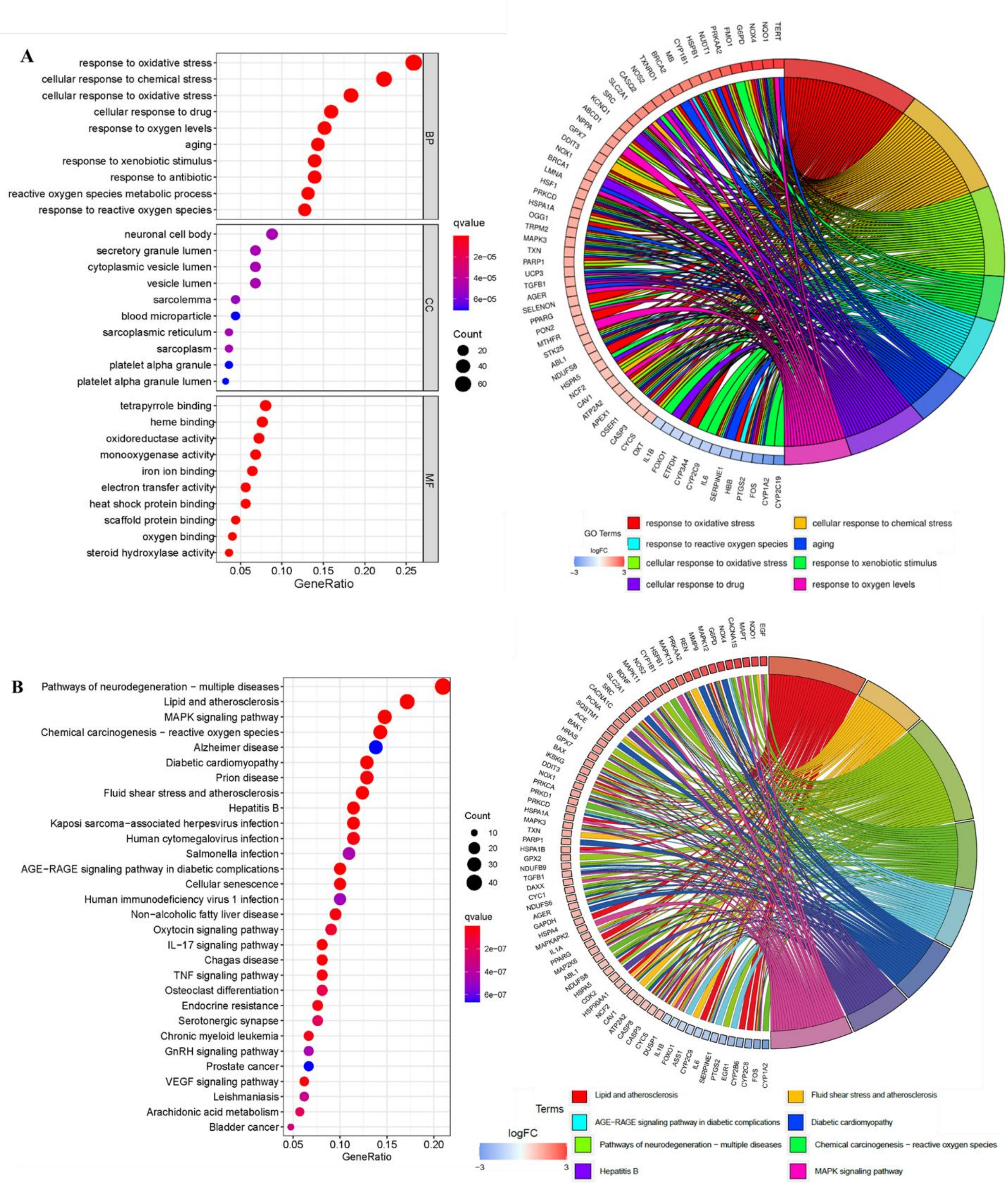

Fig. S1 Functional enrichment analysis of differentially expressed oxidative stress-related genes (DEOSGs). (A) Top 10 classes of GO enrichment terms in biological process, cellular component and molecular function(right) and circle diagram of GO enrichment analysis (left) (B) Top 30 classes of KEGG enrichment terms(right) and circle diagram of KEGG enrichment analysis (left).

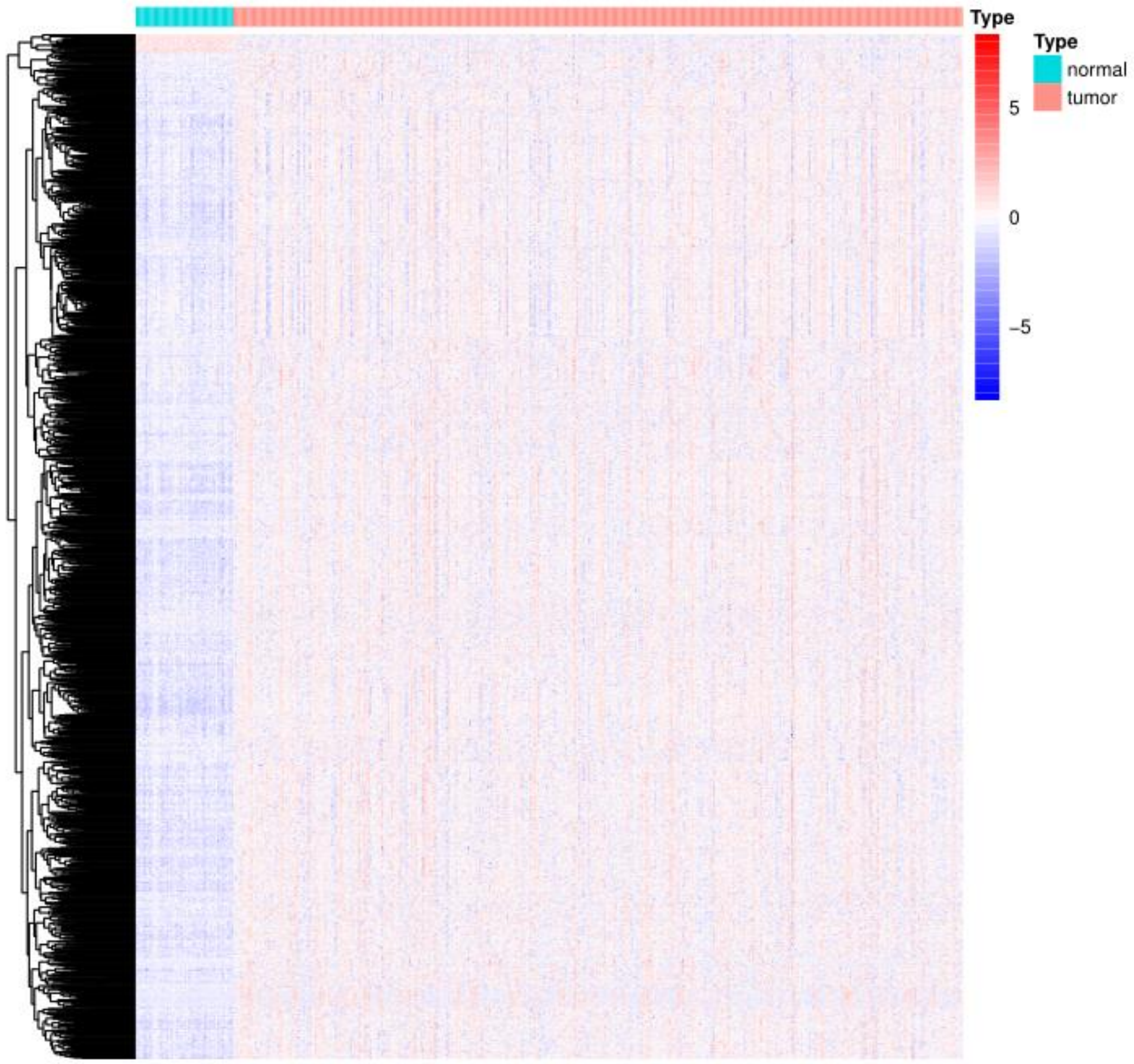

Fig. S2 The heatmap of total oxidative stress-related DElncRNAs expression pattern.

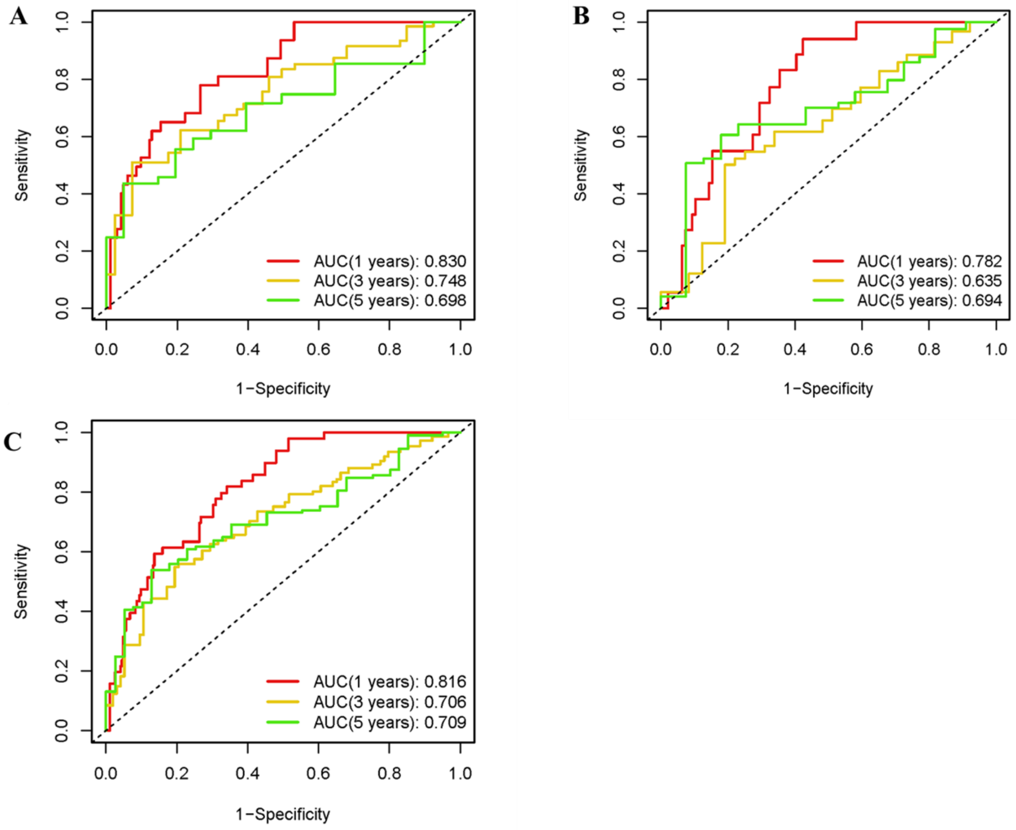

Fig. S3 The 1-, 2-, and 3-year ROC curves of the (A) training dataset, (B)testing dataset, and (C) complete dataset, respectively.

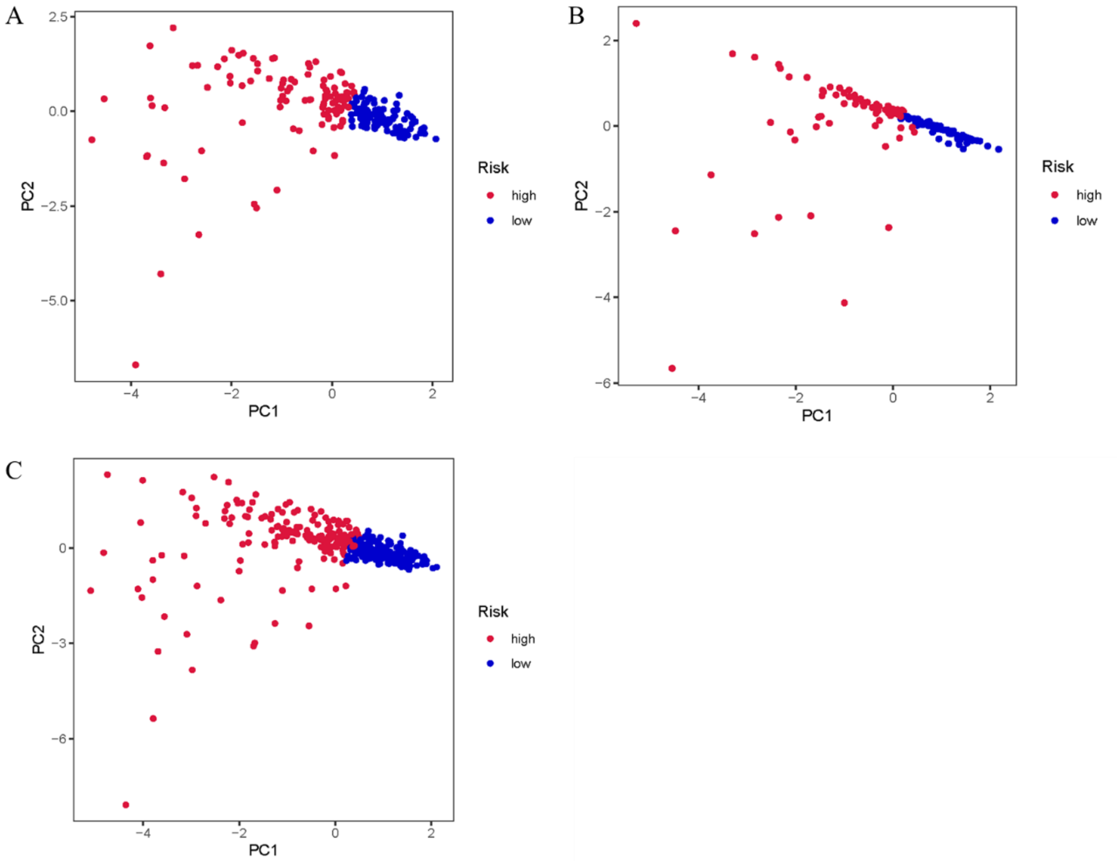

Fig. S4 PCA plot of high-risk and low-risk groups based on four oxidative stress-related DElncRNA signatures in (A) training dataset, (B) testing dataset, (C) complete dataset.

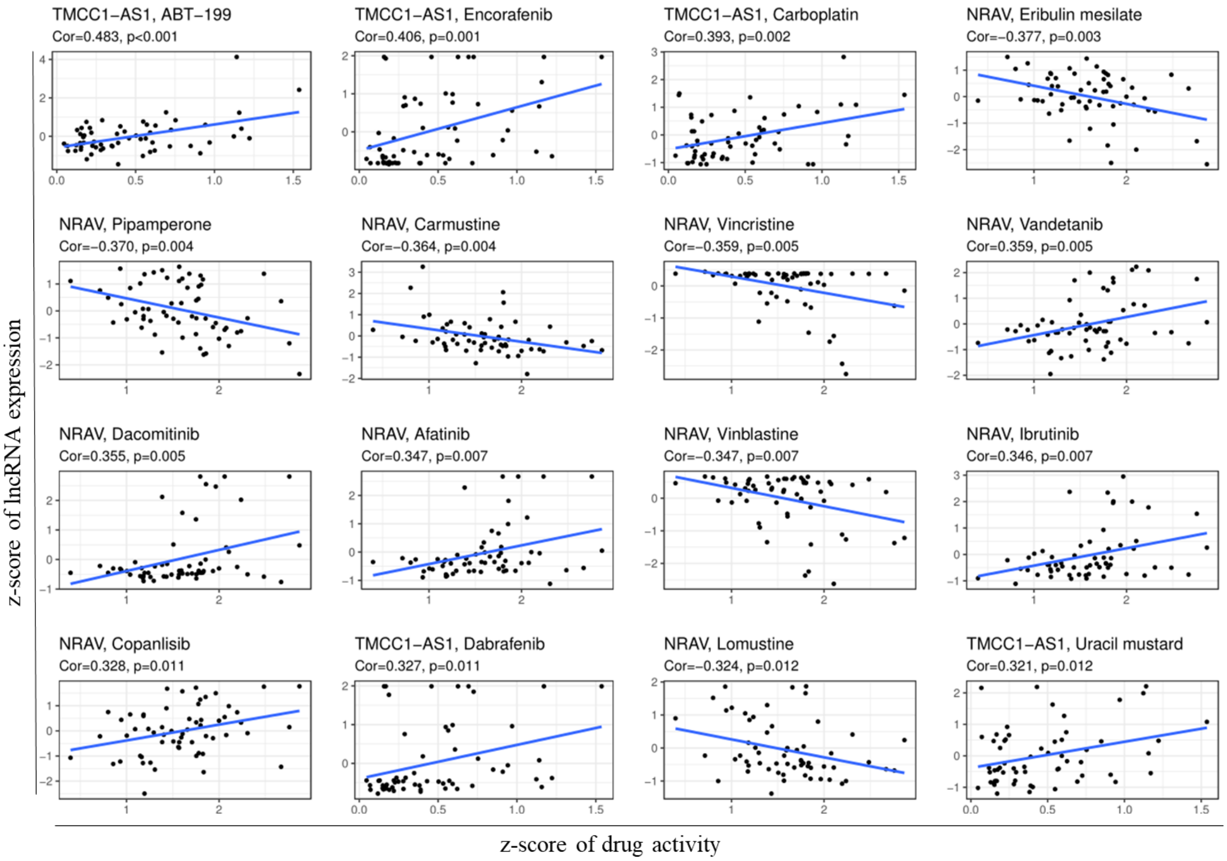

Fig. S5 Top 16 significant correlations of two oxidative stress-related DElncRNAs (TMCC1-AS1 and NRAV) and FDA-approved drugs (p<0.05).
